# Uplifting local ecological knowledge as part of adaptation pathways to wildfire risk reduction: A case study in Montseny, Catalonia (Spain)

**DOI:** 10.1007/s13280-024-02030-7

**Published:** 2024-05-25

**Authors:** Kathleen Uyttewaal, Cathelijne R. Stoof, Guillem Canaleta, Maria Cifre-Sabater, E. R. (Lisa) Langer, Fulco Ludwig, Carolien Kroeze, Pepa Moran, Isabeau Ottolini, Núria Prat-Guitart

**Affiliations:** 1 Water Systems and Global Change Group, Wageningen University, P.O. Box 47, 6700 AA Wageningen, The Netherlands; 2 Wageningen University and Research, PO box 47, 6700 AA Wageningen, The Netherlands; 3 Pau Costa Foundation, Av. Mossèn Cinto Verdaguer, 42 Esc. A Bxs 2a, 08552 Taradell, Barcelona Spain; 4 UOC, Rambla del Poblenou, 156, 08018 Barcelona, Spain; 5 Scion: New Zealand Forest Research Institute, P.O. Box 29237, Christchurch, 8440 New Zealand; 6 UPC, Carrer de Jordi Girona 31, 08034 Barcelona, Spain

**Keywords:** Adaptation pathways, Local ecological knowledge, Mediterranean, Transdisciplinary, Wildfire risk

## Abstract

Living with wildfires in an era of climate change requires adaptation and weaving together many forms of knowledge. Empirical evidence of knowledge co-production in wildfire management is lacking in Mediterranean European areas. We explored how local ecological knowledge can be leveraged to reduce wildfire risk through an adaptation pathways process in the Montseny massif and wider Tordera River watershed of Catalonia, Spain: an area stewarded through forestry and agriculture, tourism, nature conservation, and fire management. We combined different methods (e.g., a timeline and Three Horizons framework) throughout three workshops with agents of change to co-create adaptation pathways to reduce wildfire risk, integrating a historical perspective of the landscape while envisioning desirable futures. Our results showed that local ecological knowledge and other soft adaptation strategies contribute to innovative sustainable development initiatives that can also mitigate wildfire risk. The adaptation pathways approach holds much potential to inform local policies and support wildfire-based community initiatives in diverse contexts.

**Supplementary Information:**

The online version contains supplementary material available at 10.1007/s13280-024-02030-7.

## Introduction

Wildfire management is increasingly recognized as a complex social–ecological issue (Essen et al. 2023). Climate and land use change will further challenge wildfire management as more frequent and severe events create uncertain and life-threatening scenarios for fire responders and civilians (Castellnou et al. 2019). But this situation also provides an opportunity to consider diverse actors’ agency, and the dynamic quality of fire-disturbed landscapes, to create more sustainable ways of “living with fire” (Moran Núñez 2020; Stoof and Kettridge 2022). Reducing wildfire risk, therefore, requires co-management by various actors, at different scales, and different points of time before, during, and after a fire event (Kirschner et al. 2023). Transformative redistribution of knowledge and power in wildfire management, especially to Indigenous communities and other land stewards, can help reduce some of these risks (Christianson et al. 2014; Lambert and Scott 2019). Transdisciplinary approaches can bridge work between research, action and policy, providing needed tools, knowledge, and collaborations for these efforts (Brown et al. 2010). Adaptation pathways provide one such transdisciplinary approach, in which decisions in climate adaptation research and planning are implemented according to changing future conditions and the development of knowledge and policy (Werners et al. 2021a, b).

In Mediterranean Europe, widespread land use change in the twentieth century has altered the vegetation in many landscapes. At the same time, wildfire regimes are expected to intensify and become more unpredictable with climate change (Tedim et al. 2018). Meanwhile, local knowledge about the landscape is quickly diminishing due to rural abandonment and disenfranchisement, driven by rapid transitions from subsistence-oriented to global market-driven economies, industrial development, and a shift to the service sector (especially tourism) (Santamarina and Bodí 2013; Chergui et al. 2018). This combination of uncertainty, social and ecological change, and die-off of knowledge requires creativity and collaboration to address intersecting issues of global change.

Mediterranean European land and wildfire managers are transforming decades-old fire suppression policies by addressing governance gaps between communities and institutions (Wunder et al. 2021). Local communities are initiating bottom-up processes for prevention and preparedness, shifting expectations of top-down institutional intervention (Tedim et al. 2016; Ottolini et al. 2023). European-wide frameworks aim to spark fire-smart territories, and the Spanish government has developed strategic guidelines to increase ecosystem adaptation actions and community participation for wildfire risk reduction (Rego et al. 2018; CLIF 2022). This shift must be facilitated by transdisciplinary methodologies that enable community engagement and weave together different knowledge systems (Huffman 2013; Martínez-Sastre et al. 2017; Tedim et al. 2021).

Diverse localized knowledge systems are critical to understand and engage with changing wildfire regimes in many areas of the world (Norgaard 2014; Christianson et al. 2014; Stone and Langer 2015; Copes-Gerbitz et al. 2021)*.* Some of these include local ecological knowledge (LEK), traditional ecological knowledge (TEK), and traditional fire knowledge (TFK), and the terms are sometimes used interchangeably (Joa et al. 2018). All these knowledges share qualities of social transmission, rootedness in place, and constant adaptation to changing social and ecological conditions (Gómez-Baggethun and Reyes-García, 2013). We use the term local ecological knowledge (LEK) for Mediterranean European context.^1^ LEK can help improve land management, “by sustainably managing natural resources, fostering biodiversity conservation, or enhancing adaptive capacity to environmental change” (Hernández-Morcillo et al. 2014: 9). This knowledge is deeply embedded in managing agro-silvo-pastoral systems and can help maintain diverse economic and cultural activities, shaping landscape “mosaics” in rural areas (Amici et al. 2015; Guadilla-Sáez et al. 2019). However, local knowledge is in danger of disappearing in Mediterranean Europe (Huffman 2013). For instance, shepherds who once lit pastoral fires have faced sanctions and incarceration, thus eroding the knowledge base and triggering more covert and high-risk burns (Fernández-Giménez and Estaque 2012; Oteros-Rozas et al. 2013). Few European areas have institutionalized prescribed burning programs, which are strictly delegated to government agencies and allow little inclusion of wider community members. Today, fire managers, locals, and researchers recognize that the total professionalization of fire knowledge risks side-lining LEK (Coughlan 2013; Uyttewaal et al. 2023). However, only a few studies in Europe observe *how* LEK can be more actively incorporated into wildfire management in a co-productive way (Jucker Riva et al. 2018; Otero et al. 2018).

Wildfire management needs to empower local governance, and uplift local knowledge to adapt to climate change (Kelly et al. 2023; Kirschner et al. 2023). To this end, adaptation pathways present a transdisciplinary learning framework (Werners et al. 2021a, b). Adaptation pathways originated in the water management sector and have since been applied across a range of sustainability disciplines (Pereira et al. 2020; Kuiper et al. 2021; Schaal et al. 2023). A subset, called climate resilient development pathways, provides a promising transdisciplinary approach to fire risk issues, as fire risk forms part of a broader challenge of sustainable and management (Tedim et al. 2016; Otero and Nielsen 2017). This approach requires collaborative engagement between multiple actors and focuses on the past development, future aspirations, and understanding climate risks (Werners et al. 2021a, b). Further, climate resilient development pathways present a decision-orientated focus, aiming to assess and implement alternative management actions within complex social–ecological systems (SES) facing high uncertainty, and facilitate long-term sustainable development (Werners et al. 2021a, b). To our knowledge, few climate resilient development pathways focus on uplifting local knowledge as an essential component of wildfire management (Maru et al. 2014; Prober et al. 2017; Hill et al. 2020).

To this end, we sought co-constructive tools that describe historical pathways of change while illustrating the challenges and opportunities in the present and future contexts. Two such examples are timelines and the Three Horizons framework: when combined, they can reveal SES dynamics in the past, present, and future (Sharpe et al. 2016; Hill et al. 2020).

We aimed to explore how local ecological knowledge can be leveraged to reduce wildfire risk through an adaptation pathways process. For this, we combined historical perspectives and the Three Horizons methodology in a case study in Catalonia, Spain, focusing on the role of LEK to encourage adaptive and transformative actions for wildfire risk management.

## Materials and methods

### Study area

**Fig. 1:**
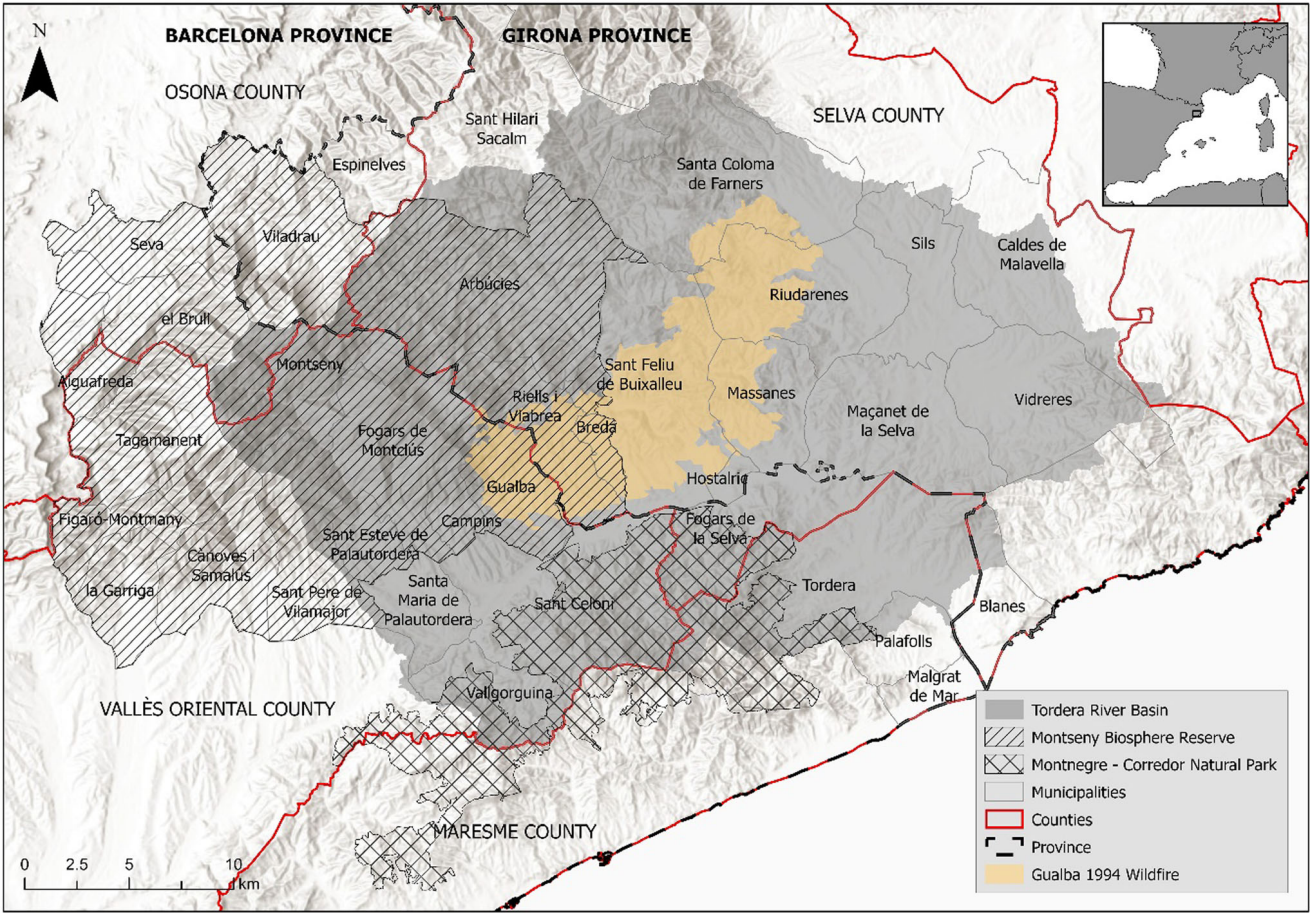
Study area of the Montseny-Tordera. Administrative boundaries include Girona and Barcelona provinces, three counties, and municipalities. Geographic features include the Tordera watershed, the Montseny Biosphere Reserve, and the Montnegre-Corredor Natural Park. The 1994 Gualba fire perimeter is shaded in yellow

We based our study 70-km NE of Barcelona, Spain, in an area encompassing the Montseny Natural Park (part of the Catalan pre-littoral range) and the wider Tordera River Basin that overlaps for a large part with the Montseny (herein referred to as Montseny-Tordera) (Fig. 1). The 55 km^2^ watershed is composed of 81% forest area, straddles two provinces, three counties, and 27 municipalities, and is home to two natural parks (Montseny and Montnegre-Corredor) and a UNESCO Biosphere Reserve (Fig. 1) (Pascual et al. 2015; Sanchez-Plaza et al. 2019). The Montseny-Tordera habitats represent most climatic zones in the Mediterranean region: sensitive “sentinel” social–ecological landscapes that provide important indicators of global change (Bonet and Vallès, 2002; Pujantell Albós et al. 2020). Human presence has shaped this landscape for at least 5000 years (Alay i Rodríguez and Zamora i Escala, 1994). The area experienced significant land abandonment in the mid-twentieth century due to economic diversification and population growth while booming industrial development and tourist attractions expanded for urban residents (Bellaubi et al. 2021). As such, landscape diversity is deteriorating due to the disappearance of peasant land use and ensuing forest expansion (Otero et al. 2015). The area also faces climatic pressure: Temperature increases have already directly affected sensitive ecosystems like European beech forests (*Fagus sylvatica*), and streamflow is projected to decline significantly (Peñuelas and Boada 2003; Bellaubi et al. 2021). Temperatures are likely to further increase and precipitation decrease (Gràcia et al. 2017). Finally, the study area experiences infrequent but severe wildfires: The last large destructive wildfire was the 11,136 ha Gualba wildfire in 1994 (GRAF 1994). It classified as a large wildfire (“Gran Incendi Forestal”) as it surpassed suppression capacity due to high winds, accumulated drought, high temperatures, relative humidity below 30%, and massive fuel continuity (GRAF 1994). Furthermore, the group of wildfire specialists in the Catalan fire department, GRAF, has classified the Gualba wildfire as a fifth-generation wildfire due to: its intense fire behavior with rapid propagation, convective behavior, high flame lengths and multiple secondary foci, the scenario of simultaneous fire incidents throughout Catalonia, and the impact it had on the interface with urban settlements (GRAF 1994; Costa et al. 2011).

**Fig. 2:**
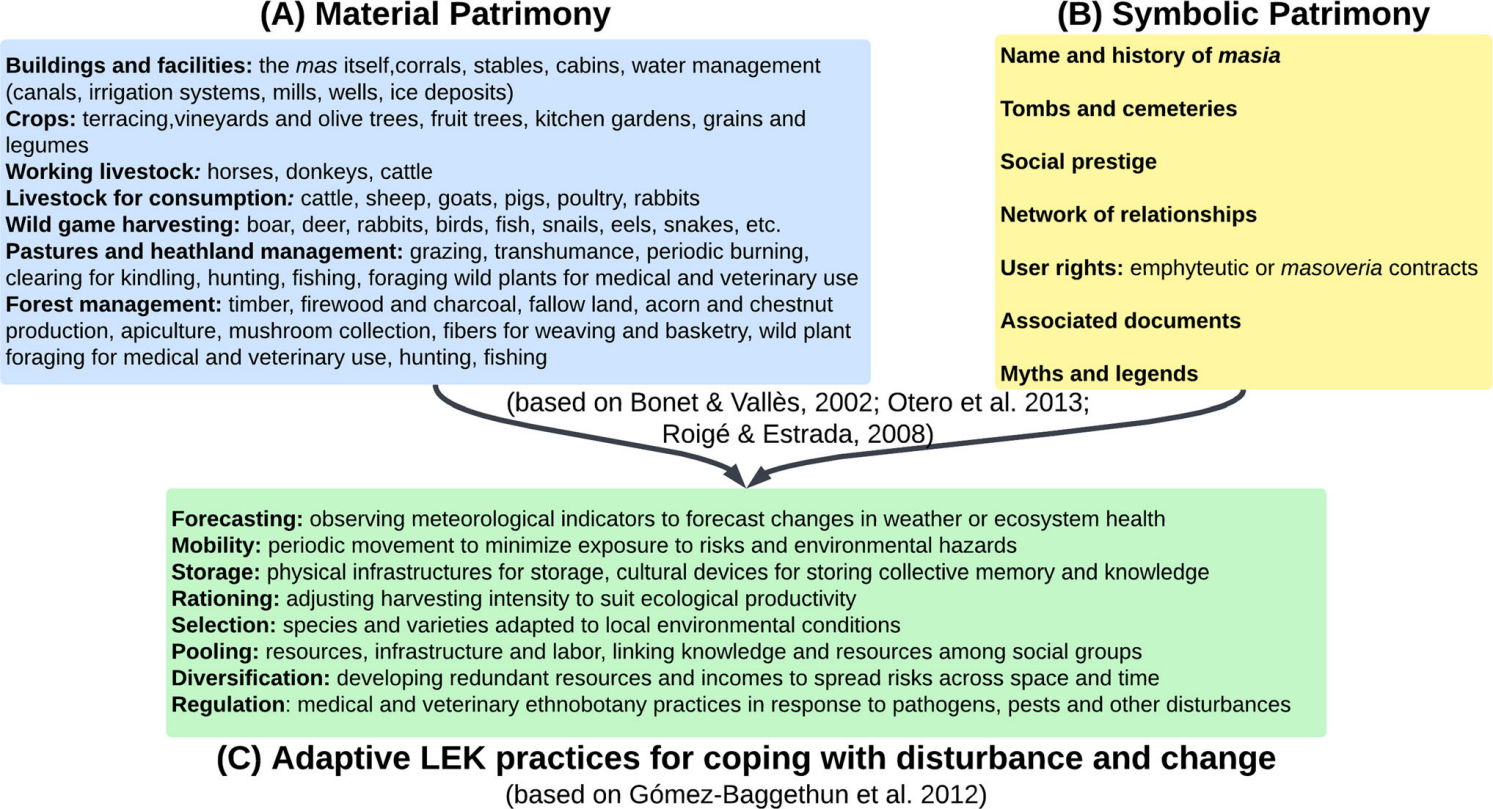
Components of LEK that pertain to the Catalan masia social–ecological system. Material and symbolic patrimony form part of wider adaptive practices for coping with disturbance and change, as developed by Gómez-Baggethun et al. (2012)

The Montseny-Tordera’s most significant LEK system has dominated the landscape use for the last thousand years: the Catalan *masia* (or farmhouse). *Masies* have created exceptional biocultural diversity and a deep sense of heritage, containing valuable insights for conserving Mediterranean landscapes under global change (Campos et al. 2014). The kinds of LEK associated with the *masia* system consist of material and immaterial patrimony such as agriculture, forest, and water management (Fig. 2) (Roigé and Estrada 2008; Bonet and Vallès, 2002; Otero et al. 2013). Much like other Mediterranean LEK systems, the *masia* system demonstrates adaptive practices for coping with disturbance and change (Gómez-Baggethun et al. 2012). While the knowledge base regarding *masia* management has greatly eroded in its application, these practices nevertheless hold important lessons for climate change (Otero et al. 2013). This is because they developed over hundreds of years, in varied conditions, and are tailored to the local context. We recognize that the *masia* LEK system may not be directly transferrable to wildfire management, and constitutes just one of the forms of land management in the area (Otero and Nielsen 2017; Rodriguez Fernández-Blanco et al. 2022). However, the institutional and socioecological legacy of *masies* over generations has shaped current private property relations and continues to influence agricultural and forestry practices which hold key lessons for sustainable wildfire *prevention* strategies (Roigé and Estrada 2008; Otero et al. 2013). For these reasons, we focus our study on examining how LEK in the Montseny-Tordera can form part of adaptation for wildfire risk management, in tandem with other forms of more technical knowledge, as held by the GRAF and forest planners, for instance.

### Participant selection

We invited 58 participants with rich case-specific knowledge based on the snowball technique and purposive sampling (Suri 2011; Leventon et al. 2016). These participants were considered as *agents of change*: holding embodied knowledge through diverse life experiences, with their involvement forming part of a personal and collective transformative process (details in Appendix S1) (Westley et al. 2013; Charli-Joseph et al. 2018). Such participants included local associations of shepherds and forest owners, regional and local wildfire risk planners from provincial, county and city councils, LEK holders (e.g., *masia* inhabitants, traditional agriculture, and forestry practitioners), associations of forest defense (ADFs), sustainable and educational tourism initiatives, GRAF operatives (wildfire specialists in the Catalan Fire Department), Montseny Natural Park employees, university researchers, and non-governmental organizations (NGOs) dedicated to agroecology and fire ecology knowledge exchange. Participants who held LEK were not necessarily identified as such due to their age, but due to their livelihoods and shared intergenerational knowledge.

Out of 58 invitations, 26 participants attended at least one workshop, 17 of whom participated recurringly or contributed via personal communications (more information in Appendix S1). While our outreach strategy aimed to represent diverse ages and genders, we did not implement participation quotas or explicitly gather demographic information from the volunteer participants. However, we observed that the majority were aged over 40, at least ten were aged above 50, and 70% identified as men.

### Workshop process

Three workshops took place between July 2022 and March 2023 in the Montseny-Tordera (Table 1), to: (1) generate a historical timeline, then envision an idealized future, (2) describe possible pathways to achieve this vision through back-casting, and (3) reflect on the pathway outcomes to consider preferred next steps. These workshops were based on a climate resilient development pathways framework (Werners et al. 2021a, b) that considers improving livelihoods, social and economic well-being, and responsible environmental management—key components for increasing wildfire resilience in rural Mediterranean areas (Verkerk et al. 2018). We combined this with a “trajectories of change” approach, taking a historical perspective to identify system dynamics and potential lock-ins through a timeline activity (Werners et al. 2021a, b). In total, the sum of these approaches (the timeline activity, the Three Horizons approach, and the reflections on the outcomes) generated our adaptation pathways process.

**Table 1:** Outline of workshops, their dates, details, and attendance by participants (facilitators and observers not included)

Date and event	Title	Research approach	Specific activities	Research outputs	Participants
June 2021–May 2022	Preliminary interviews	Understanding local social context	Semi-structured interviews with key participants and additional experts on aspects of the local social context, current fire adaptation strategies, and current values/perspectives on fire preparedness (Uyttewaal et al. 2023)	Semi-structured interviews	19
May 2022 Public kick-off event	Past, present, and future of wildfire in Montseny-Tordera	Setting the context, networking	Round table discussion of the past, present, and future of wildfire in the Montseny area. Field trip included to observe historical wildfires in the landscape and consider potential future wildfires	Purposive sampling and snowball technique through contacts	30
July 2022 Workshop 1	Where have we come from? Where do we want to go?	1. Timeline (trajectories of change) 2. Visions (“Horizon 3” of Three Horizons)	1. Co-created timeline of important changes in SES 2. Future local wildfire risk scenarios modeled & discussed 3. Visioning activity: even with a future more at risk of wildfires, what would the landscape look like ideally? (Horizon 3)	1. Co-created timeline outputs 2. Co-created vision outputs	9
September 2022 Workshop 2	How do we get there?	Back-casting exercise (based on Horizon 3 from anterior workshop, to complete Horizon 1 and 2)	Back-casting exercise: 1. What current activities support this plural vision of the future? (Horizon 3) 2. What current activities hinder this vision of the future? (Horizon 1) 3. What activities in the mid-term could help us get to where we want to be? (Horizon 2)	Co-created Three Horizons outputs	17
March 2023 Workshop 3	Reflections	Reflect on completed adaptation pathways	Reflections on the adaptation pathways: What have we learned through this process? Which actions seem most interesting, actionable, influential, longer-term, and why?	Annotated adaptation pathways	15

#### Workshop 1: Timeline and visioning

Nine participants first co-created a timeline (trajectories of change) outlining important changes in the local socio-ecological system, aiming to: (a) describe primary drivers of change in the SES; (b) connect social and ecological changes, especially in local knowledge systems and wildfire risk; and c) highlight local knowledges as part of the process of change. Participants were divided into two groups: One group demonstrated ownership of LEK (e.g., traditional agricultural practitioners, extensive livestock managers, *masia* inhabitants, and a university professor specialized in local SES), while the other group exhibited a high level of administrative and technical knowledge (e.g., regional planners and fire risk technicians, a fire ecology NGO, local forestry business, and an ADF). A general discussion then aimed to join these timelines, to find connections between events and discuss the most significant drivers of change. We grouped results into eight classes, defined according to the previous work (Uyttewaal et al. 2023): broader historical processes, agricultural and forestry sectors, local knowledges, ecological changes, political and legislative changes, touristic and industrial sectors, urban development, and “generations” of fire behavior (Castellnou et al. 2019).

After the timeline activity, participants received an information session on current and expected future fire behavior in the Montseny-Tordera, in order to ground subsequent discussions within current and projected wildfire scenarios (details in Appendix S1). The following visioning activity formed part of the Three Horizons approach (Horizon 3), where actors with high agency can work with uncertain futures in creative ways while also retaining important features from the present (details in Appendix S1). Horizon 1 represents a “business as usual” or current system that needs to change, Horizon 3 represents an ideal future system, and Horizon 2 represents a transformative middle zone that leverages change to get from Horizon 1 to Horizon 3 (Sharpe et al. 2016). Our adaptation pathways were created by following the Three Horizons approach. Participants were asked to imagine an ideal fire-resilient landscape in 2050, based on four sectors established through prior research (Uyttewaal et al. 2023): *What would an ideal fire-resilient landscape look like to you in 2050, in the following categories: forest management, agriculture and extensive grazing, tourism, and urban development?* Landscapes represent a cultural image, and visualizing can help uncover its meanings, embrace multiple perspectives, and work with people’s aspirations and values, all of which are tenants of co-productive research (Cosgrove 1998; Sharpe et al. 2016; Chambers et al. 2022). Discussions were held in mixed subgroups to encourage maximum dialog among different sectors. Participants were asked to think specifically and creatively, creating a collage of their results and sharing their visions in a general discussion, representing Horizon 3. After analysis, this activity generated 35 vision components.

#### Workshop 2: Back-casting

The visions (Horizon 3) generated in Workshop 1 formed the basis for the back-casting workshop (Workshop 2). Now that participants knew “where they came from and where they would like to go,” we focused on how they could get there through defining specific action points. New participants could add their own values to the visions if they were not represented by prior participants. In four subgroups, 17 total participants discussed existing good practices in need for upscaling (part of Horizon 3), along with current malpractices that need to be phased out over time (part of Horizon 1). Then, they discussed the transitions, innovations, and tensions to be addressed to make these more desirable landscapes possible (Horizon 2). Subgroup results were discussed in a plenary session, generating 28 possible actions after analysis (or Horizon 2 points) (details in Appendix S1).

#### Workshop 3: Reflections on the pathways

In Workshop 3, the 28 pathway actions were presented, which 15 participants then “scored” and discussed: *Which actions are most interesting to you, which ones could be immediately implemented, which are suitable for longer-term implementation, and which ones could act as key influencers for other actions?* Participants reflected first individually, and then in small group discussions: Each person annotated a copy of the pathways to accompany the discussion. These scorings were used to guide the debates (details in Appendix S1).

### Analysis

After each workshop, participants’ outputs (sticky notes and vision collages) and discussions (recordings) were transcribed and doublechecked with observers (details in Appendix S1). Data were organized through matrix and tabulation methods, then analyzed through thematic analysis in NVivo (Miles and Huberman 1984; Saldaña, 2013), and finally translated from Catalan to English.

Our analysis of the timeline outputs aimed to describe the context and uncover primary drivers of change in the SES. The outputs of the Three Horizons activity (the visions from Workshop 1 and back-casting results from Workshop 2) were analyzed to uncover the themes and sectors involved, and if and how each action point developed by workshop participants related to LEK. Finally, our analysis of the annotated pathways (Workshop 3) aimed to distinguish participant preferences for the action points and to consider how these preferences related to uplifting LEK in the territory.

In order to focus on LEK as part of wildfire resilience, we first had to define what LEK consists of on a local level (Fig. 2), and then decipher mechanisms that would directly support LEK longevity and expansion. For this, our research team cross-referenced each statement with Fig. 2 as well as prior work in the area detailing local socioecological heritage (Otero et al. 2013), and then categorized the findings with Gavin & Tang’s framework of threats and potential conservation responses to LEK (2016). Here, they encouraged research and documentation, community-based activities, education and awareness, capacity-building, and policies and legislation. These LEK conservation themes are not mutually exclusive, however, and several of the proposed action points could have indirect linkages to LEK. Though we recognize this analysis is not exhaustive or exclusive, we aimed to identify as direct a relationship as possible between the workshop outputs and Tang & Gavin’s LEK conservation framework. We further recognize the complexity and pitfalls of creating boundaries on what LEK is or is not, as it is a dynamic body of knowledge that is constantly adapting (Reyes-García et al. 2014). Given the novelty of this work, we consider this analysis to provide a starting point to consider *how* LEK can form part of wildfire risk reduction in European Mediterranean contexts. Results were communicated back to participants via two graphics: (1) an illustration of the “visions” (Fig. 4) generated by landscape architecture master students in an external collaboration at Universitat Politècnica de Catalunya (UPC) based on the themes presented in Table 2, and (2) an easy-to-read matrix illustrating 28 distinct actions that represent important transitional potential to achieve these goals (Uyttewaal et al., in review).

**Table 2:** Visions of an idealized fire-resilient landscape in the future of the Montseny-Tordera area, featuring their relevance to upholding local ecological knowledges (LEK), and the ways in which these may be conserved, as developed by Tang and Gavin (2016)

Components of future visions	Direct link to LEK	LEK conservation theme				
		Research/documentation (6)	Community-based activities (11)	Education & awareness (7)	Capacity-building (18)	Policy/legislation (6)
**Forest management**						
Agroforestry mosaic that is diverse and dynamic in space and timing	X		X		X	
Traditional livelihoods are restored and relevant (shepherding, gardening, tending chestnut groves, etc.)	X		X		X	
Ecosystem perturbations are introduced (herbivory and fire) with modern criteria (i.e., prescribed burning, prescribed grazing…)	X	X	X		X	
Recovered open spaces (i.e., meadows and pastures)	X		X		X	
Larger “rings” of land managed around *masies*: homes, gardens, pasture, and forests	X		X		X	
Traditional water sources are well managed (such as wells and traditional aquifer systems)	X		X		X	
Diversified local products (non-wood products, high-quality wooden structures for public use…)	X		X		X	
New forest industries for chemical and industrial use	–	–	–	–	–	–
Better balance between producing renewable energy and capturing carbon	–	–	–	–	–	–
Dynamic equilibrium between clear-cut areas, young forests, mature forests, and conservation areas	X		X		X	
Fair and sustainable water management	X				X	
Forests are accompanied to adjust to new climate conditions through strategic planning	–	–	–	–	–	–
Multiple uses of the landscape are prioritized, while recognizing that not all activities are compatible in the same space (e.g., hunting, hiking, biking, grazing, forestry, and motorists)	–	–	–	–	–	–
**Extensive livestock & agriculture**						
Diversity of livestock (sheep, goats, cattle, horses, and donkeys) and rotation of fields according to seasons	X	X	X		X	
Restored connectivity between pastures on mountain crest areas	X				X	
Diversity of local production and demand (meats, dairy products, wool, skins, soaps, etc.)	X		X	X	X	
Ecosystem services from livestock are valued and compensated (recovering and maintaining open space, generating biodiversity, and managing fuel load)	X	X		X	X	
Young people are incentivized and trained in sustainable livestock production	X			X	X	
New technologies implemented for strategic rotation (GPS, mobile electric fences, etc.)	–	–	–	–	–	–
Some areas reserved seasonally for livestock grazing (better territorial planning)	X				X	X
**Tourism**						
Natural parks communicate better about social–ecological components of the landscape	X	X		X		
Model of educational tourism that encourages activities that steward the landscape	X	X		X		
Local products are highly valued, widely accessible, and economically viable for producers	X			X	X	X
Functions in support of primary production: creates active links between restaurants, other consumers, and land managers	X					
Public has high awareness that the landscape is dynamic and changing	X			X		
**Urban development**						
Architectural guides in place for using local primary material and conserving the cultural patrimony of the area	X	X			X	X
*Masies* are restored and inhabited with modern criteria (e.g., access to water/electricity/internet, solar panels, with permission to construct additional structures if necessary)	X		X		X	X
Necessary infrastructures in place for primary sector (i.e., butcheries, small elaboration facilities for products, appropriate warehouses for machinery/storage, etc.)	X					X
Better infrastructure for remote working in rural areas: better public transportation, health, and education access	X					X
New housing developments come with environmental risk already integrated: change to fire-resistant materials and fire-resilient development planning	–	–	–	–	–	–
Detailed fire-resilient planning takes into account: fire types, wind, topography, vegetation, clear evacuation routes, and meeting points	–	–	–	–	–	–
**Other (global change and governance)**						
Reduced reliance on fossil fuels	–	–	–	–	–	–
More democratization and accurate representation in local decision-making	–	–	–	–	–	–
More social cohesion and cooperation between sectors help place pressure on administrative change of restrictive policies on small farmers and foresters	–	–	–	–	–	–

The dash (-) indicates that the component does not relate directly to upholding LEK

## Results

### Timelines (trajectories of change)

**Fig. 3:**
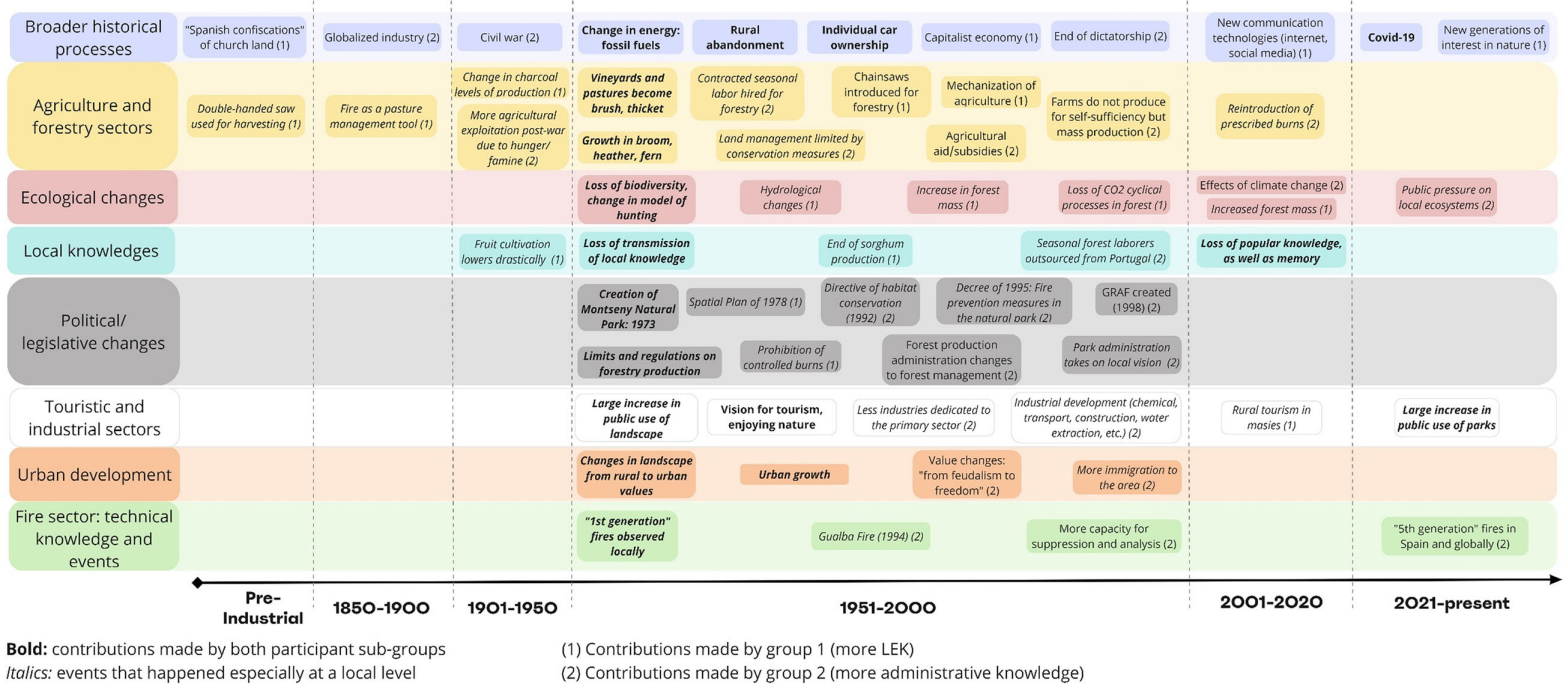
Timeline of system dynamics in the Montseny-Tordera River Basin from preindustrial periods to the present, created by participants in Workshop 1. Objects in bold signify events that both participant subgroups shared. Objects in italics are events that happened especially on a local level. Objects in the same row do not indicate direct linear relationships between objects, nor do they indicate precise moments in time

The participants’ combined knowledge illustrated changes in energy systems, urban development, patterns of land ownership and management, and rural abandonment (Fig. 3) which all shaped the landscape and current wildfire risk over several decades.

Participants contributed to the timeline primarily based on their own lifespans, as noted due to their average age range. Notably, they pointed to 1950–2000 as a period where strong local and global change drivers convened (Fig. 3). For example, they described how globalized forestry industries (due to mechanization and chainsaw development) led to intensive forest management on larger properties, while large-scale rural abandonment also led to an increase in shrub and forest mass, homogenizing vegetation, and depleting the LEK base. Meanwhile, participants shared that global conservation movements also inspired local action, and legislation led to creating Montseny Natural Park in 1973 and a spatial plan in 1978 protecting natural areas while also severely limiting human activity. Participants reported that drivers of change such as globalized economies and fossil fuel industries exacerbated rural depopulation: People no longer used wood or charcoal for heating, many looked for better economic opportunities in towns and cities since small-scale farming was disincentivized, and more productive forestry in other areas of the newly formed EU outcompeted local markets. And while ecological consciousness aided in protecting natural areas, participants indicated that this “protection” of natural areas also accelerated the loss of LEK in rural populations as many traditional management activities ceased. While participants did not elaborate in great detail on the kinds of LEK that were present, the discourse was clearly tied to *masia* systems. Participants observed that the trajectory of biodiversity and LEK loss began most acutely in the 1960s: They observed how the sum of these complex changes in social, political, and economic configurations locally and globally resulted in a much more homogeneous and flammable landscape, materializing locally for the first time with the disastrous 1994 Gualba wildfire.

### Visions (Horizon 3)

**Fig. 4:**
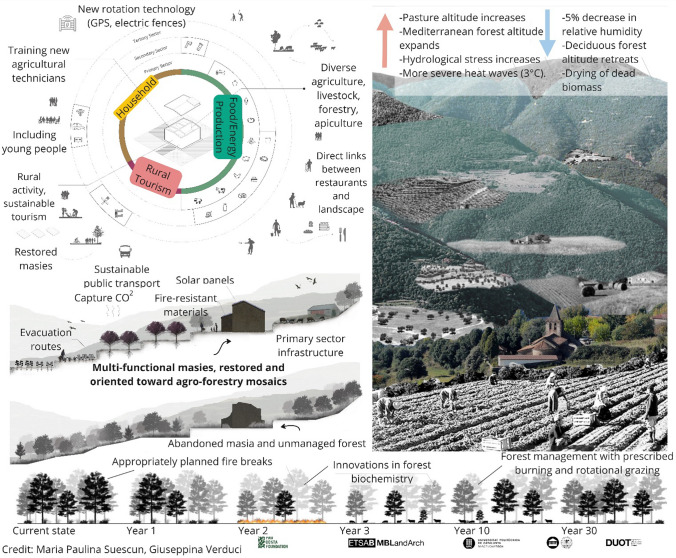
Representation of the visions of a future fire-resilient landscape in the Montseny-Tordera area, as developed and illustrated by masters students in landscape architecture at the Universitat Politècnica de Catalunya. The image indicates “rings” of land management around masies, juxtaposes an image of a managed and unmanaged masia, considers the effects of perturbations like prescribed fire and herbivory, and demonstrates elements of an idealized future scenario despite expected changes in climate and vegetation

The main themes emerging from participants’ ideal futures considered forest management, extensive livestock and agriculture, tourism, urban development, global change, and governance. This exercise produced 35 vision components (Table 2), which were then illustrated and used in posterior workshops as a visual reference tool for our “desired horizon” or Horizon 3 (Fig. 4). Post-workshop analysis revealed that 24 components (69%) related to Tang and Gavin’s (2016) LEK conservation themes (Table 2). In the visions, LEK could inform various innovations, such as: in science and research (*ecosystem perturbations are introduced with modern criteria [i.e., prescribed burning and prescribed grazing]*); economic development (*diversified local products [non-wood products and high-quality wooden structures for public use]*); urban development (*architectural guides in place for using local primary material and conserving the cultural patrimony of the area*); and sustainable land use (*model of educational tourism that encourages activities that steward the landscape*). The non-LEK vision components (40%) considered more international developments, especially in forest management and global change. For instance, imagining *new forest industries for chemical and industrial use*, a *better balance between producing renewable energy and capturing carbon*, and *reduced reliance on fossil fuels* would require coordination and investment from actors far beyond the local landscape. While many aspects of these future visions do not connect directly to supporting LEK, it demonstrates that participants placed importance on representing plural values in the landscape.

Participants did not focus as much on which specific kinds of LEK to maintain or revive, much like the prior timeline activity. Instead, they looked to deeper underlying support systems such as educational initiatives, financial input to maintain infrastructures, and specific political actions aimed to increase the viability of rural livelihoods, thus ensuring that knowledge can get passed on and remain relevant in the future. The categories of LEK conservation most represented in the visioning exercise included the growth of community-based activities (11 components; 31%) and local capacity-building (18 components; 51%, Table 2). The categories are not mutually exclusive, since most vision components engage with multiple LEK conservation themes. For instance, *ecosystem services from livestock are valued and compensated*, would require research, education, and capacity-building. Another, *diversity of local production and demand,* would require community activities, education, and capacity-building. A related component where *local products are highly valued, widely accessible, and economically viable for producers,* would also require additional policy and legislative support.

### Pathways to achieve wildfire-resilient landscapes

Our analysis revealed that 19 of the 28 actions developed by participants can preserve and revive LEK in the study area (68%, Table 3). Of these 19 actions that can directly support LEK, 11 (58%) scored of high interest to the participants in the final workshop to prioritize due to their actionability, long-term effects, and role as key influencers that can help catalyze other actions (Table 3). In fact, 11 of the total 16 highest-scoring actions (69%) were oriented toward LEK conservation. High-scoring actions that did not directly relate to LEK included diverse approaches from social sectors, bioeconomy, planning and administration, and fire management. For instance, participants valued “low-hanging fruit” directly related to fire management: *better regulating access to certain roads and parking lots in times of risk*. More complex administrative actions were also highly regarded, such as: *improving forest planning at a higher than private property scale, coordinating between existing planning and administrations *(*e.g., fire management, forestry, water, biodiversity, *etc*.*) (Table 3).

**Table 3:** Adaptation pathway actions (Column II—outputs analyzed from the back-casting workshop) are interpreted for several criteria

I LEK conservation theme					II Adaptation pathway actions	III Participant preferences			
Research/documentation	Community activities	Education/awareness	Capacity-building	Policy/legislation	Participants’ highest-ranking actions	Most interesting	Longer-term results	Immediate action	Key influencers
	X	X	X		(S) Support education and a local training school based on traditional activities, livestock, forestry, and other land management aspects		X		X
		X			(S) Generate environmental education programs with field trips to get to know the local landscape		X		X
	X		X		(S) Facilitate more contact and collaboration between different producers of various products in the area			X	
				X	(S/P) Accompany the primary sector in bureaucratic and administrative issues (e.g., an office that helps prioritize collective initiatives, a concrete figure that stimulates meeting points and takes actors’ values in account in decision-making, promotes initiatives between local administrations and private properties to manage landscapes)	X			
–	–	–	–	–	(S) Take advantage of climate change awareness to motivate small forest owners to manage their tracts	–	–	–	–
			X	X	(B) Promote locally produced renewable energy: firewood and derivatives	X	X		X
			X	X	(B) Create associations and make forest management more economically accessible by scale			X	
			X	X	(B/P) Facilitate access to work (payment for ecosystem services—water management, biodiversity, reducing wildfire risk, etc.)	X			
–	–	–	–	–	(B) Foster the attraction of young people: give support to new ideas and livelihoods of sustainability	–	–	–	–
X			X		(P) Support species in their adaptation: chestnuts, cork oaks, beech trees, etc.		X		
				X	(P) Reform spatial plans, so that the "protection" of a space is not always linked to the limitation of permitted activities		X		
–	–	–	–	–	(P) Improve forest planning at a higher than private property scale, coordinate between existing planning and administrations (e.g., fire management, forestry, water, biodiversity, etc.)	X	X		
X				X	(P) Give more importance to open spaces (like pastures) in fire prevention plans	X			
–	–	–	–	–	(P) Create sustainable forest management plans: estimate harvests that can maintain production, conserve biodiversity, and limit CO2 emissions	X			
			X	X	(E) Improve the availability of water for extensive livestock			X	
–	–	–	–	–	(F) Better regulate access to certain roads and parking lots in times of risk			X	
					Participants’ lower ranking actions				
X	X	X			(S) Highlight “lighthouse” initiatives: groups or organizations that practice sustainable land management and show potential to adapt				
	X		X		(S) Facilitate better communication between existing formal and informal knowledge networks				
	X		X		(S) Support “land bank” initiatives and provide consulting (put owners in contact with people who want to live in *masies*, manage abandoned land, etc.)				
X		X			(S) Disseminate and visualize success stories of local production: show models and numbers of viable management				
			X	X	(S) Increase services in isolated areas and “micro-villages”: schools, health, commerce				
	X	X	X		(B) Promote labeling campaigns for local products				
–	–	–	–	–	(B/P) Connect local discourses and resources to global change discourse (create an agency to facilitate access to aid from the EU and local agencies in the transition from fossil fuels)				
X			X		(P) Develop criteria for architectural coherence and integration with the landscape using local materials: wood, dry stone, etc.				
	X		X		(E) Restore ancient irrigation channels and water sources, domestic, and public water tanks				
–	–	–	–	–	(E/P) Regulate the extraction and over-use of groundwater (e.g., private bottling plants)				
–	–	–	–	–	(F) Recover wastewater for wildfire prevention and suppression (e.g., Comunitat Valenciana)				
–	–	–	–	–	(F) Evaluate and analyze wildfire risk areas before building more homes and developments				

We first organize these actions as they fall under broad themes of more social actions (S), bioeconomy-oriented actions (B), planning and administrative actions (P), environmental management (E), or wildfire risk management (F). Then, we consider if and how these actions abide by LEK conservation themes (Column I—ex-post analysis), as developed prior by Tang and Gavin 2016. Participant preferences for these actions are represented in Column III as outputs analyzed from the learning workshop

Participants preferred adaptation strategies that were more social and educational, that uplifted local bioeconomy activities around food and energy production, and that reshaped territorial planning. Out of the highest-scoring LEK actions (Table 3), four involve social actions, five involve planning and administrative action, three involve bioeconomy oriented actions, and one involves environmental management. For instance, *supporting education and a local training school based on traditional activities, livestock, forestry, and other land management aspects* scored highly among participants as ways to create longer-term change and provide key influences over other adaptation strategies. It also scored highly as a LEK conservation action that would improve community capacity, create more opportunities for community-based activities, and promote broader education and awareness around local human–nature relationships.

Another action point, *promoting locally produced renewable energy: firewood and derivatives*, nods to the area’s rich history in forestry production while also looking forward toward sustainable forest management in the context of climate change. Derivative products include biomass and pellets for efficient wooden stoves and biochar for renewable agroforestry initiatives. Participants stated that this kind of action can create long-term economic and opportunities in vegetation management and technological innovation, and could play a key role in influencing other adaptation actions due to economic incentive (such as implementing sustainable management plans and strengthening forest owner associations). Indeed, some forest owners shared frustration on the over-reliance on subsidies while large forestry companies in northern Europe flood the international market. This kind of action could help fixate knowledge and resources in the area, by: (1) building more resources and economic capacity in the forestry sector and (2) incentivizing policies and legislations at regional and international levels in order to better regulate crowded forestry markets. This can support LEK conservation indirectly by incentivizing the primary forestry sector, thereby maintaining and expanding the existing knowledge base.

On the other hand, other participants voiced caution that leaning too heavily on re-commodifying forestry production could lead to maladaptive overexploitation: *“In reality, if we look back, [energy production was sustainable] because it was a different lifestyle. But with the current population, trying to implement a renewable energy system might not be that sustainable”* (Workshop 3). This kind of action demonstrates transformative potential in local economies and international policies on sustainable energy transitions, though it must be approached with appropriate policies so that sustainable forest management is not subject to abrupt shifts in markets and resource availability.

Lastly, a preferred planning-oriented action like *giving more importance to open spaces (like pastures) in fire prevention plans* reveals opportunities in research, documentation, and policy changes to support the regulating ecosystem services that pastures (and shepherds) provide, and which could help fixate LEK associated with this sector through legislative support.

## Discussion

### The past and future of wildfire risk management

Our results demonstrated two main trends: While LEK has experienced significant decline, diverse participants still hold and highly value LEK when considering more wildfire-resilient futures. The timeline activity (Fig. 3) shared consistent findings with other studies in Spain and Portugal, revealing that LEK has experienced sharp decline since the 1950s in the area, affecting biodiversity, influencing the homogeneity, and fuel loading of the landscape (Otero et al. 2013; de Oliveira et al. 2023). This exercise also illustrated that global societal and ecological change have shaped wildfire risk locally (Otero and Nielsen 2017). The continuity of LEK will only be ensured if efforts are made to restore and adapt this knowledge system in a changing world (Hernández-Morcillo et al. 2014).

Participants decidedly valued LEK-related actions in our pathways: 11 out of 16 high-scoring pathway actions tied directly to LEK conservation. In fact, a majority of the vision and pathway components (69% each, Tables 2 and 3) relate directly to LEK conservation in some way. The pathways demonstrated several areas where planning and administrative changes are needed, but they also showed that change can come from many other sectors and strategies (such as social, educational, and economic initiatives). While the actual implementation of these strategies reaches beyond the scope of this study, this finding highlights the actionable intentions of local participants. This helps counteract the narrative that “passive” communities in the Mediterranean areas often await intervention from institutions rather than implementing bottom-up initiatives (Tedim et al. 2016). Furthermore, it demonstrates how LEK can help lead some of these changes.

Meanwhile, additional components not directly linked to LEK (Table 3) suggest that there are many complementary ways to promote wildfire risk reduction and sustainable development. LEK represents an important part of pluralistic values in the landscape: It does not exclude other innovations, such as new technologies in forestry, transitions away from fossil fuels, or incorporating wildfire risk criteria into new urban developments. Indeed, LEK itself is a dynamic process that includes innovations and does not necessarily belong to specific people (Reyes-García et al. 2014). These advances can simultaneously coexist in the territory without negating the other; this acceptance of pluralism is central to processes of adaptation (Colloff et al. 2021).

The visioning activity (Fig. 4 and Table 2) furthermore revealed that activities supporting LEK can also create more resilient landscapes to wildfire when combined with other risk management tools and innovations. For instance, if we consider the five themes of a fire-resilient landscape as promoted by Newman Thacker et al. (2023), the visions generated by participants in the workshop contribute directly to acceptance and use of fire in the landscape (e.g., through prescribed burning), broader landscape management (e.g., through agro-silvo-pastoral practices), deep-seated community engagement (e.g., formal and informal education activities), loss avoidance (e.g., through integrated wildfire risk planning before creating new urban developments), and recovery (e.g., maintaining intergenerational connections in the area, accompanying primary tree species in their adaptation to climate change, etc.). These themes of fire resilience are especially relevant as fire activity is projected to increase with climate and land use change throughout Catalonia, and simultaneous fire events threaten to cause multiple civil emergencies due to limited response capacity (Castellnou et al. 2019).

### “Soft” adaptation and generalized resilience

Our results also exposed numerous opportunities for “soft” adaptation strategies that elevate LEK in sustainable land management, with wildfire risk reduction as an added value. Such “soft” adaptation approaches include investments in “improving technical, organizational and social capacities of administrative and social systems to respond to climate-related stress” (Dolsak & Prakash 2018: 326). These efforts require co-productive efforts between a wide range of actors on different scales. In the present study, examples included place-based (in)formal education, knowledge exchanges across siloes of expertise, supporting cooperative associations among producers, and policies that uplift sustainable and diverse food and energy sectors (Table 3). Some initiatives could be easily implemented locally (such as fostering land-based education initiatives). Other actions require coordination at regional, national, and international levels, such as energy transitions away from fossil fuels and utilizing sustainable forest-derived energy sources. While workshop participants recognized the importance of mitigating wildfire risk directly (e.g., vegetation management and integrating risk in urban planning), they prioritized further-reaching soft adaptation strategies. This reflects many participants’ livelihoods in sustainable land management, and it also echoes calls from resilience scholars encouraging broader movements of “generalized resilience” to shocks (including wildfires), rather than hyper-focusing on “specified resilience” that may unintentionally create maladaptive responses to broader climate change and development issues (Carpenter et al. 2012). For instance, traditional post-wildfire revegetation efforts have resulted in poorly-suited (e.g., invasive non-native, not drought-adapted, mono-specific) ecosystem trajectories (Sample et al. 2022). Our findings do not imply that direct wildfire risk reduction efforts (such as municipal risk plans, managing home ignition zones, fuel management, and creating firebreaks) are less important: Rather, their effectiveness can be enhanced when paired with local values toward sustainable development. Indeed, recent research by Ascoli et al. (2023) demonstrate that wildfire prevention must consider local specificities and strengthen the role of traditional activities that contribute to fuel management, in order to elevate their “cost efficiency.” Furthermore, the authors argue that many bottom-up initiatives for wildfire risk prevention can meet the goals of EU Green Deal policies. Specifically, they reviewed and encourage programs that: recognize wildfire prevention as an ecosystem service, integrate different sectoral policies (such as forestry, agriculture, nature conservation, energy production, and tourism), cluster public and private land partnerships, use diverse treatment approaches, value local agro-forestry products, and support strong social engagement (Ascoli et al. 2023). Coincidentally, each one of these strategies has also emerged in our co-produced adaptation pathways. This demonstrates that suggestions from Ascoli et al. apply on a local level, and it also speaks to the robustness of our co-productive approach and its applicability on a broader Mediterranean European scale.

### Inclusion and imagination

Several of the workshop activities fostered a sense of inclusiveness, shared values, and imagination. For instance, the visioning activity focused on local knowledge holders’ desires, combined with input from other agents of change from varied sectors. Notably, participants largely reached toward similar goals: This speaks to the many shared values in the area, even among diverse sectors. Prior adaptation projects in the Montseny-Tordera also reveal highly shared values in the area despite the diversity of contributing participants (Verkerk et al. 2017; Otero et al. 2018). While these results may be a result of potential sample bias (Section “Limitations and recommendations for further research”), they also provide further insight. These shared values contributed to a sense of cohesion and output ownership since the beginning of the process and facilitated onboarding of people who were not able to attend the first visioning exercise. In another example, the participants also built a shared understanding of widespread societal transformation in the timeline activity, especially from the 1950s onwards, based on their lived experience and that of their elders. By observing these dynamics on paper, it also allowed participants to imagine that significant societal change in the coming decades can and will occur due to climate change, social change, and transitioning economies away from fossil fuels. Indeed, the ability to imagine other realities is necessary in transdisciplinary work to move beyond past approaches that have failed to address complex societal problems (Brown et al. 2010).

This encouraged much creative thought around the possible visions of the landscape, and the ensuing back-casted actions (Table 3) included a wide range of incremental and more transformative actions to achieve this. For example, while some adaptation actions (Table 3) could be achieved with simpler public engagement methods (e.g., *generate environmental education programs with field trips to get to know the local landscape*), others would require national and international reform. These included actions centered around sustainable energy production (*promote locally produced renewable energy: firewood and derivatives*), more cooperative economic models (*create associations and make forest management more economically accessible by scale*), more opportunities for collaborative governance (*accompany the primary sector in bureaucratic and administrative issues*), and redistribution of private property (*support “land bank” initiatives and provide consulting).* Indeed, global academic literature and political discourse point toward the need for both incremental and transformative shifts toward planetary sustainability and ecological justice (IPCC 2022). It is especially significant that these shifts are considered necessary even in a highly localized case study such as this.

This process’s outcome may have been influenced by a lack of major fire disasters in the area since the 1990s. A feeling of relative safety may have allowed participants to think more openly and creatively. At the same time, participants considered the effects of a multi-year drought, contributing to a sense of urgency during the discussion. Our process took place during a moment of heightened awareness, though paired with a feeling of safety and agency, which may have helped avoid “rigidity traps” to innovation during our discussions (Butler and Goldstein 2010).

### Limitations and recommendations for further research

Our process faced several limitations that are not new in transdisciplinary collaborations. These included navigating biases and power relations, placing boundaries on LEK systems, and implementing actions beyond the scope of the project. Firstly, our participant selection resulted in a few biases: We deliberately aimed to engage with agents of change and local knowledge holders, rather than explore all the possible visions from actors with varied forms of power in the area. Additional perspectives exist in the area, including those of large private water and forestry industries, tourism geared toward urban consumers, and conservation efforts espousing strict non-intervention, which we acknowledge were not included in the process. As such, next steps in this co-constructive process could encourage wider profiles of participants, thereby developing more variety between visions and pathways, and illustrating more complex dynamics happening in the landscape. Broader participant diversity (based on categories such as gender, wider socioeconomic, and ethnic backgrounds) could additionally contribute to a wider range of histories, values, visions, and subsequent pathway results. Further work may also consider the effects of imbalances in political and economic power that highly influence the study area and have led to deep-seated social tensions, especially in the sectors of tourism, conservation, and primary production. In our case, facilitated inclusion techniques helped to navigate some tensions to best represent voices (especially LEK holders) that are not often given a platform. In these collaborative settings, it is important for researchers and participants to consider tradeoffs: who gains and who loses, who has access to decision-making, capital, and other resources. These processes are often fraught and contested, and LEK conservation should not be treated as a cure-all solution or be used to reinforce exclusionary attitudes about who belongs in a rapidly changing landscape (Blythe et al. 2018). In the present study, it was furthermore challenging to define what LEK actually consists of in the area. Given waves of abandonment and shifting values, as well as the politics of placing boundaries on a knowledge system that is dynamic and constantly adapting, too-rigidly defining LEK may risk tokenizing certain forms of knowledge while ignoring others (Sharma 2021).

As other transdisciplinary collaborations have observed (Mauser et al. 2013; Leemans and Fortuin 2023), the constraints of institutional support, short funding cycles, and small research-action teams informed our approach, which led to rich co-produced discussion and relationship-building but few tangible results in the short term (continued in Uyttewaal et al., in review). For creating longer-lasting impact, an additional further research step could explore social and ecological thresholds and tipping points as other adaptation pathways studies have done (Haasnoot et al. 2013), in order to further refine strategic interventions with low risk of maladaptation and which could directly inform local policies and fundraising mechanisms. While the role of our research was not to implement the actions we defined, we encourage future community engagement work through boundary-spanning organizations like the Pau Costa Foundation, which can directly collaborate with many local participants.

## Conclusion

This paper explored how LEK can be leveraged in wildfire risk management through an adaptation pathways process. With local agents of change in the Montseny-Tordera area (Catalonia, Spain), we combined a historical perspective on trajectories of change with the Three Horizons to center LEK as a key component of historical change and future sustainable development. We found that the timeline method (Fig. 3) enabled participants to understand interrelated system dynamics and acknowledge radical changes in the landscape, and the Three Horizons enabled participants to think creatively about their futures (Fig. 4 and Table 2) while maintaining a deep rootedness to place (Table 3). Throughout the workshop process, LEK was framed as a key aspect of both the past and the potential future of the system, helping to sustain LEK-related actions as a high priority when discussing adapting to living with fire. The results also demonstrate LEK’s suitability to be situated in the future socio-ecosystems according to change agents’ values in the area, and this knowledge system is compatible with other innovative and transformative actions as part of plural pathways to change. In short, this process allowed consideration of both the past and the future of wildfire management, encouraged specific actions toward adaptation and resilience, while fostering an inclusive and imaginative environment. Collaborations are needed across sectors and scales to keep LEK alive, to pursue sustainable development pathways, and to reduce wildfire risk. The adaptation pathways approach demonstrates how actors can find common points of interest across all three issues and learn from one another, which is the topic of further work. Collective visioning and back-casting provide a first important step to inform local policy and wider action, and as such is highly viable in other territories to generate deeper connections between sectors working toward more desirable, sustainable, knowledge-rich, and fire-resilient futures.

## Supplementary Information

Below is the link to the electronic supplementary material.

Supplementary file1 (PDF 201 kb)

## Footnotes

1 Knowledge, practices, and beliefs regarding ecological relationships gained through long-term personal observation and interaction with local ecosystems, and shared among a population (Charnley et al. 2007).
