# Supplementary material for: Uplifting local ecological knowledge as part of adaptation pathways to wildfire risk reduction: A case study in Montseny, Catalonia (Spain)
Source: Ambio. 2024 May 25;53(10):1433–53. doi: 10.1007/s13280-024-02030-7 (PMC11383912; doi:10.1007/s13280-024-02030-7)
Supplement: Supplementary file 1 — Supplementary file1 (PDF 201 kb) [file 13280_2024_2030_MOESM1_ESM.pdf]

Ambio

Supplementary Information

This supplementary information has not been peer reviewed

Appendix S1: Supplementary Methodological Background

2.3 Participant selection

These individuals possessed a) broad social networks through their work in collectives or associations, education, and local administrations, b) enthusiasm to participate and willingness to learn from others c) understanding of wider social-ecological processes d) a sense of place attachment, and e) understanding of LEK in the area, as well as innovative approaches to managing the territory.

This more restricted number of participants was partly due to scheduling conflicts, given the participants' volunteer efforts. Additionally, a screening process (due to divergent perspectives between some sector representatives) ensured that productive dialogues could occur without personal tensions changing the collaborative atmosphere (Chambers et al., 2022).

The research team included 3 facilitators as well as 6 observers. While facilitators led the workshop activities, observers aided in each subgroup by keeping discussions on subject and taking notes.

2.4.1 Workshop 1: timeline and visioning

This group visualization maps different events shaping an issue, highlighting complex system interactions that help participants reflect on interconnected events (Brouwer & Brouwers, 2017). This can help participants create a shared and plural understanding of the main drivers and current SES state, therefore identifying important areas of engagement (Bosomworth et al., 2018).

Climate change informational session

Following the timeline activity, participants received an informational session describing current and potential future fire behavior in the Montseny-Tordera area. This information was modelled on landscape components (surface fuel models, elevation, orientation, slope and canopy fuels) and weather scenarios (temperature, relative humidity, wind and vegetation water stress). The climate change scenario considered the diagnosis, impacts and vulnerabilities to climate change predicted in the Montseny-Tordera area to adjust future fuel models and vegetation water stress (Campeny & Ferrer, 2016). This information was shared with participants by experts from a local fire ecology NGO in order to ground the following activities within the current and potential ecological context. Visualizing a future wildfire disturbance regime, not a traumatic wildfire event, can help shift narratives and emotions away from fear-based reactions (Moran Núñez, 2020).

2.4.2 Workshop 2: back-casting

A total of 77 sticky notes resulted from the four mixed subgroup discussions. These were grouped according to themes, accounting for overlaps, and were then analyzed and condensed by the research team.

2.4.3 Workshop 3: reflections and learning

Participants were asked to choose their top 5 actions for each question: *Which actions are most interesting to you, which ones could be immediately implemented, which are suitable for longer-term implementation, and which ones could act as key influencers for other actions?*

Given the natural flow of the discussion groups, we refrained from scoring action points quantitatively since not all participants adhered to the scoring guidelines and new information emerged during discussions.

2.5 Analysis

Additional information that emerged verbally during subgroup conversations and plenary sessions were recorded and transcribed. Guided by transcriptions and trained observers' notes for each sub-group, we organized entries, combined duplicate messages, and clarified inputs with the observers. Data were summarized and tabulated, then underwent thematic analysis.

References:

Bosomworth, K., Scott, H., Wilson, J., Brunt, K., Pitfield, C., Johnson, F., & Brown, G. (2018). *Exploring "Adaptation Pathways" planning through an NRM lens: Insights from two exploratory case studies*. RMIT University. ISBN: 978-0-9953791-3-8

Brouwer, H., & Brouwers, J. (2017). *The MSP Tool Guide: Sixty Tools to Facilitate Multi-Stakeholder Partnerships*. https://doi.org/10.4324/9781315146096-2

Campeny, R., & Ferrer, E. (2016). *Estudi dels efectes del canvi climàtic en el Montseny: diagnosi, impactes i vulnerabilitats*.

Chambers, J., Wyborn, C., Klenk, N., Ryan, M., Serban, A., Bennett, N., Brennan, R., Charli-Joseph, L., Fernández-Giménez, M., Galvin, K., Goldstein, B., Haller, T., Hill, R., Munera, C., Nel, J., Österblom, H., Reid, R., Riechers, M., Spierenburg, M., … Rondeau, R. (2022). Co-productive agility and four collaborative pathways to sustainability transformations. *Global Environmental Change*, *72*. https://doi.org/10.1016/j.gloenvcha.2021.102422

Moran Núñez, F. J. (2020). *Paisajes Disruptivos: La representación de los incendios forestales en el paisaje mediterráneo* [Universitat Politècnica de Catalunya (UPC)]. https://doi.org/10.5821/dissertation-2117-335419
